# Role of the Beta Catenin Destruction Complex in Mediating Chemotherapy-Induced Senescence-Associated Secretory Phenotype

**DOI:** 10.1371/journal.pone.0052188

**Published:** 2012-12-18

**Authors:** Dipanjan Basu, Miguel Reyes-Mugica, Abdelhadi Rebbaa

**Affiliations:** Department of Pathology, University of Pittsburgh and the Children’s Hospital of Pittsburgh of UPMC, Pittsburgh, Pennsylvania, United States of America; The University of Texas Health Science Center, United States of America

## Abstract

Cellular senescence is considered as a tumor suppressive mechanism. Recent evidence indicates however that senescent cells secrete various growth factors and cytokines, some of which may paradoxically promote cancer progression. This phenomenon termed senescence-associated secretory phenotype (SASP) must be inhibited in order for anti-proliferative agents to be effective. The present study was designed to determine whether the β-catenin destruction complex (BCDC), known to integrate the action of various growth factors and cytokines, would represent a suitable target to inhibit the activity of SASP components. For this, we carried out experiments to determine the effect of drug-induced senescence on secretion of SASP, β-catenin transactivation, and the relationship between these processes. Moreover, genetic and pharmacological approaches were used to define the implication of BCDC in mediating the effects of SASP components on cell migration and resistance to drugs. The findings indicate that drug-induced senescence was associated with expression of various Wnt ligands in addition to previously known SASP components. Beta catenin transactivation and expression of genes implicated in epithelial-mesenchymal transition (EMT) also increased in response to drug-induced SASP. These effects were prevented by Pyrvinium, a recently described activator of BCDC. Pyrvinium also suppressed the effects of SASP on cell migration and resistance to doxorubicin. Together, these findings provide insights on the potential role of BCDC in mediating the effects of drug-induced SASP on cancer cell invasion and resistance to therapy, and suggest that targeting this pathway may represent an effective approach to enhance the activity of current and prospective anti-cancer therapeutics.

## Introduction

Cellular senescence is a signal transduction program that leads to irreversible proliferation arrest in response to endogenous or exogenous stressors [1]. It was initially considered as a manifestation of the overall decline in activity associated with aging of somatic cells [2], however subsequent studies have shown that certain therapeutic agents induce a premature form of senescence [3], [4], [5], suggesting that this cellular process may be exploited for the development of approaches to suppress tumor growth. This view was however quickly challenged by the finding that, although senescent cells do not proliferate, they remain metabolically active and able to secrete soluble factors, some of which may paradoxically promote cancer metastasis and resistance to therapy [5], [6], [7]. In fact, it is now recognized that among the prominent senescence-associated changes in gene expression, there is a robust increase in the synthesis and secretion of numerous cytokines, chemokines, growth factors and proteases [6], [8], [9], a phenomenon termed senescence-associated secretory phenotype (SASP) [10]. The DNA damage response (DDR) was initially introduced as a causative factor [11] however subsequent studies have demonstrated that epigenetic alterations [12] and activation of MAP kinases [13] may also induce SASP, suggesting a multifactorial aspect of the underlying mechanisms.

Components of SASP such as TGFβ, EGF, Wnt ligands, IL8, and IL6, to cite just a few, are known for their ability to promote tumor progression through the inhibition of apoptosis [6], induction of epithelial-mesenchymal transition (EMT) [14] and/or resistance to therapy [7]. Therefore, the action of these secreted factors must be inhibited in order for anti-proliferative agents to be effective. Towards this goal, previous effort resulted in the identification of corticosteroids as potential candidates to suppress the synthesis of certain cytokines and growth factors implicated in SASP [15]. Likewise, the use of MAP kinase inhibitors also reduced SASP activity and enhanced cancer cell response to drugs [13]. However, due to the multitude of signaling pathways activated by SASP, a preferred molecular target must integrate the action of many if not all of them. We reasoned that such target could be the beta catenin destruction complex (BCDC) since it has been shown to function at the convergence of signaling pathways initiated by growth factors, cytokines, Wnt ligands, sonic hedgehog, and G protein-coupled ligands [16]. Critical elements of this complex include GSK 3β, casein kinase I alpha (CKIα), adenomatous polyposis coli (APC) and Axin [17], [18]. In the absence of extracellular stimuli, non-phosphorylated (active) GSK 3β acts in coordination with CK1α to phosphorylate protein substrates, rendering them susceptible to proteasomal degradation [19]. In contrast, phosphorylation-mediated inhibition of GSK 3β at specific sites results in the stabilization of these substrates. β-catenin is one of the most relevant and widely described substrates of BCDC. Its stabilization results in nuclear translocation and complex formation with the TCF/LEF transcription factors, leading to transactivation of genes implicated in EMT, metastasis and resistance to therapy [20], [21], [22]. Up to now, the role of β-catenin or the associated destruction complex in mediating the action of SASP has not been described.

The present study was designed to investigate the role of SASP as a possible mechanism by which certain cancer cells evade the cytotoxic action of chemotherapy. In particular, we determined the effects of drug-induced SASP on activation of β-catenin signaling and the consequences of targeting this process on cell migration and resistance to therapy. Our findings suggest that drug-induced SASP may represent a ubiquitous cellular response to cancer therapy and provide insights on the central role of BCDC as a potential target to inhibit this process and enhance the effectiveness of current and prospective anti-cancer therapeutics.

## Materials and Methods

Human melanoma (WM115, WM266, and SK-Mel28), breast cancer MCF-7, colon cancer SW480, and neuroblastoma SKN-SH cell lines were purchased from ATCC (Rockville MA). The 293 kidney cells were obtained from Clontech (Mountain View, CA). Dulbecco’s Modified Eagle’s Medium (DMEM), MEM, RPMI, Horse serum and fetal bovine serum (FBS) were obtained from BioWhittaker (Walkersville, MD). The following drugs and reagents were obtained from the companies cited: The Super TopFlash reporter construct [23] from Addgene (Cambridge, MA); Doxorubicin, rapamycin, XAV939, pyrvinium and antibody to beta-Actin from Sigma-Aldrich (St. Louis, MO); TGF beta from Invitrogen (Carlsbad, CA); Antibodies to p21WAF1 and p53 from Santa Cruz Biotechnology (Santa Cruz, CA); Antibodies to Zeb1, Twist and β–catenin and Cyclin D from Cell Signaling Technology (Danvers, MA); secondary antibodies conjugated to horseradish peroxidase from Jackson Immunoresearch Lab Inc. (West Grove, PA); Enhanced chemiluminescence reagents (ECL) from Thermo Scientific (Rockford, IL); Immobilon-P transfer membrane for Western blots from Millipore (Bedford, MA). Reagents for DNA transfection were obtained from Life Technologies (San Diego, CA).

### Cell Culture and Transfections

Melanoma and breast cancer cells were cultured in MEM supplemented with 10% FBS as described by the supplier. Colon cancer cells were maintained in RPMI supplemented with 10% FBS, the 293 cells were cultured in DMEM supplemented with 10%FBS penicillin/streptavidin and non-essential aminoacids (Life Technologies, San Diego, CA ), and SKN-SH cells were maintained in DMEM supplemented with 10%FBS penicillin/streptomycin. Transfections were carried out in 6 well plates using a lipofectamine kit (Life Technologies, San Diego, CA ) as described by the manufacturer. Briefly, 3 µg of DNA were mixed in 100 µl of transfection solution containing 90 µl of serum free culture medium and 10 µl lipofectamine. After 20 min incubation at room temperature, the mixture was added to the wells and incubated for 5 hours. The medium was then replaced with a new one before the inhibitors were added to the corresponding wells and incubated for an additional 24 hours. Protein extracts were harvested and processed for either Western blot or luciferase assay as described below.

### Western Blot

Proteins were extracted from cells cultivated in monolayers using 100 µl of lysis buffer (50 mM HEPES pH 7.4, 150 mM NaCl, 100 mM NaF, 1 mM MgCl_2_, 1.5 mM EGTA, 10% glycerol, 1% Triton X100, 1 µg/ml leupeptin, 1 mM phenyl-methyl-sulfonyl-fluoride). For nuclear and cytoplasmic fractionation [24], cells were re-suspended in 400 µl of buffer A (10 mM HEPES pH 7.9; 10 mM KCl;0.1 mM EDTA; 0.1 mM EGTA; 1 mM DTT; 0.5 mM PMSF). The mixture was incubated for 30 min on ice, then 25 µl of a 10% solution of Nonidet NP-40 was added and the homogenate centrifuged for 30 sec. The nuclear pellet was re-suspended in 50 µl of ice cold buffer B (20 mM HEPES pH 7.9; 0.4 M NaCl; 1 mM EDTA; 1 mM EGTA; 1 mM dithiothreitol; 1 mM PMSF). The samples were then rocked at 48C for 30 min. Following centrifugation (12000 g for 5 min), protein concentration was determined in the supernatant. Equal quantities of protein were separated by electrophoresis on a 12% SDS-PAGE gel and transferred to Immobilon-P membranes. Proteins of interest were identified by reaction with specific primary and secondary antibodies linked to horseradish peroxidase and detected by chemiluminescence.

### Q-PCR

Total RNA was extracted using the RNeasy mini kit (Qiagen, Santa Clarita, CA).The first-strand cDNA was synthesized according to manufacturer’s instructions using ThermoScript RT-PCR system (Life Technologies, San Diego, CA). Gene expression was measured by real-time PCR using the Maxima Syber green Master Mix (Fermentas, Glen Burnie, MA) on ABI 7500 instrument (Applied Biosystems, Carlsbad, CA). Q–PCR primers are reported in Table 1. Gene expression was normalized to that of GAPDH used as internal control.

**Table 1:** Primers used in QPCR.

Gene	Forward primer 5′-3′	Reverse primer 5′-3′
Wnt 3	GGAGAAGCGGAAGGAAAAATG	GCACGTCGTAGATGCGAATACA
Wnt 3a	CCTGCACTCCATCCAGCTACA	GACCTCTCTTCCTACCTTTCCCTTA
Wnt 4	GATGTGCGGGAGAGAAGCAA	ATTCCACCCGCATGTGTGT
Wnt 5a	GAAATGCGTGTTGGGTTGAA	ATGCCCTCTCCACAAAGTGAA
Wnt 5b	CTGCCTTTCCAGCGAGAATT	AGGTCAAATGGCCCCCTTT
Wnt 7b	CCCGGCAAGTTCTCTTTCTTC	GGCGTAGCTTTTCTGTGTCCAT
Wnt 10a	GGCAACCCGTCAGTCTGTCT	CATTCCCCACCTCCCATCT
EGF	CAGGGAAGATGACCACCACT	TTCCCACCACTTCAGGTCTC
IGF1	CCCCACTCACCAACTCATAG	GGTATTTGGGGCCTTTATGT
IL1α	ATCAGTACCTCACGGCTGCT	TGGGTATCTCAGGCATCTCC
CSF2	CTTCCTGTGCAACCCAGATT	CTTCTGCCATGCCTGTATCA
HGF	ATTCACTTGCAAGGCTTTTG	CAAAAAGCTGTGTTCGTGTG
β-Catenin	TGAGGACAAGCCACAAGATTAC	TCCACCAGAGTGAAAAGAACG
SMA	AGTTACGAGTTGCCTGATGG	GAGGTCCTTCCTGATGTCAA
FGF23	TGGGTTAGGTTTTCTGTGGA	AAGAATTTCCAAGGGGATTG
ICAM1	TTTTCTATCGGCACAAAAGC	AATGCAAACAGGACAAGAGG
IFN-γ	TCCCATGGGTTGTGTGTTTA	AAGCACCAGGCATGAAATCT
IL6	ATGCAATAACCACCCCTGAC	GAGGTGCCCATGCTACATTT
MMP-2	TTGACGGTAAGGACGGACTC	ACTTGCAGTACTCCCCATCG
VEGF	AGACACACCCACCCACATAC	TGCCAGAGTCTCTCATCTCC
GAPDH	GAGTCAACGGATTTGGTCGT	TTGATTTTGGAGGGATCTCG

### Top-Flash Assay

The Super TopFlash reporter (referred thereafter as TopFlash) which contains 8 TCF binding sites was used to measure β-catenin TCF transactivation [23]. To evaluate the specificity of this reaction, the FopFlash construct where the TCF binding sites are mutated was used. These plasmids were transfected transiently into cells using the lipofectamine kit as follows: 3 µg of DNA were mixed in 100 µl of transfection solution containing 90 µl of serum free culture medium and 10 µl lipofectamine. After 20 min incubation at room temperature, the mixture was added to the wells and incubated for 5 hours. The medium was then replaced with a new one before the inhibitors or conditioned medium (CM) from dells exposed to drugs were added to the corresponding wells. After incubation for an additional 24 hours, the cells were lysed and the extracts used as a source of luciferase. As a control, the FopFlash plasmid containing a mutated sequence for TCF binding was used. Transfection efficiency was measured by using a similar DNA construct in which the luciferase is substituted with EGFP (Addgene, Cambridge, MA). The percent of transfected cells was determined using a fluorescence microscope. All experiments were performed in triplicates and data is presented as average ± SE.

### Collection of Conditioned Medium (CM)

Near confluent cultured WM 115 melanoma cells grown in 25 cm^2^ flaks containing MEM and 10% FBS at 37°C in 95% air/5% CO2, were exposed to doxorubicin for 24 hours. The cell monolayer was then washed three times and placed in fresh medium for 2 hours to allow elimination of intracellular doxorubicin. The cells were then placed in 5 ml of new MEM for an additional 24 hours to allow secretion of SASP. The medium was harvested and centrifuged at 1000×g for 10 min to remove residual cells and debris. The supernatant was collected and used as conditioned medium (CM) containing putative SASP factors.

### Senescence-Associated β -Galactosidase (SA-β-Gal) Staining

Cells were seeded into 24-well plates and 24 h later, drugs were added and incubated for 4 days. The SA-β-Gal staining was performed as described previously [25]. Cells were fixed for 5 min in 3% formaldehyde and then washed three times with PBS. The fixed cells were then incubated at 37°C in a solution containing 40 mM citric acid (pH 6.5), 5 mM potassium ferrocyanide, 5 mM potassium ferricyanide, 150 mM NaCl, 2 mM MgCl2, and 1 mg/ml of 5-bromo-4-chloro- 3-indolyl β -D-galactopyranoside. After 24 h of incubation, the cells were visualized using bright field microscopy and pictures taken.

### MTT Assay

Cells were incubated in a 96 well plate with doxorubicin at varying concentrations for 96 h. Viable cells were quantitatively estimated by a colorimetric assay utilizing MTT, as described previously [26]. MTT (10 µl of 5 mg/ml solution) was added to each well of the titration plate and incubated for 4 h at 37°C. The cells were then solubilized by the addition of 100 µl of 10% SDS/0.01 M HCl and incubated for 15 h at 37°C. The optical density of each well was determined in an ELISA plate reader using an activation wavelength of 570 nm and reference wavelength of 650 nm. The percentage of viable cells was determined by comparison with untreated control cells.

### Rhodamine Uptake and Efflux

Rhodamine-123 uptake was evaluated by incubating the cells with 10 mg/ml of rhodamine for 30 min at 37°C in the presence or absence of pyrvinium (125 nM). The culture medium was then removed and cells washed twice with PBS before measure of intracellular localization of rhodamine-123 as described previously [25]. For the efflux experiment, cells were washed with PBS after rhodamine uptake and incubated in fresh medium without dye for an additional 60 min, either in the presence or absence of pyrvinium. Cells were then visualized and photographed under fluorescence microscopy (excitation, 480 nm; emission, 560 nm).

### Statistical Analysis

Graph data is presented as mean ± standard error (SE). All analyses were performed using Student *t* test. *P*<0.05 was considered statistically significant. Statistical calculations were performed with SPSS 16.0 for Windows (SPSS, Chicago, IL, USA).

## Results

### SASP and Wnt Ligands as Consequences of Cancer Cell Exposure to Drugs

**Figure 1:**
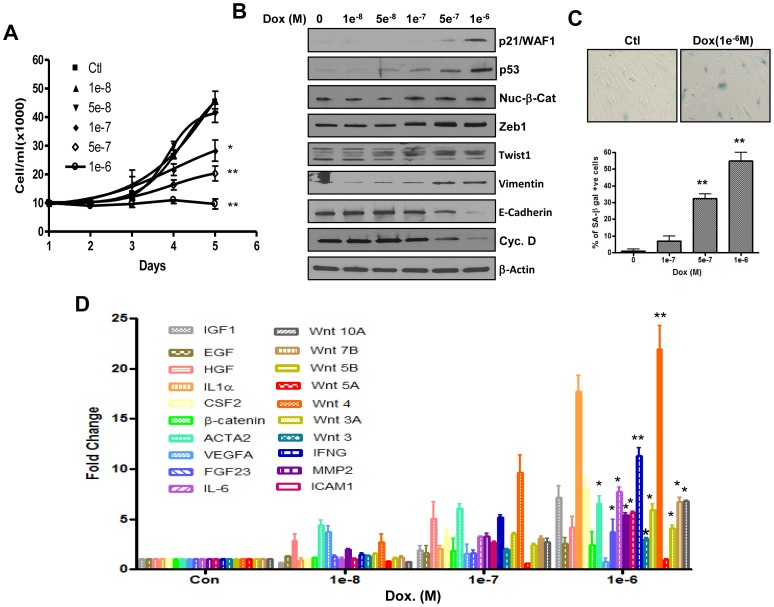
Drug-induced senescence is associated with secretion of Wnt ligands among other components of SASP. Panel A. Doxorubicin-induced proliferation arrest was determined using melanoma cells 115 exposed to the drug at the indicated concentrations for 5 days and counted every day. The data represent average ±SE of three determinations. Panel B. Western blot showing the expression of cell cycle (p53, p21/WAF1 and cyclin D) and EMT genes (Zeb1, twist1, Vimentin and E-cadherin) in the absence and the presence of doxorubicin. Panel C. SA-beta Gal staining in control and cells treated 1 µM doxorubicin viewed by bright field microscopy. Panel D. mRNA expression levels of various growth factors, cytokines and Wnt ligands in response to cellular exposure to doxorubicin. Gene expression was normalized to that of GAPDH and represented in fold change compared to control non-treated cells. Each bar represents the average of three determinations ±SE. Statistical significance is shown for drug-treated cells versus control (*p<0.05, **p<0.001).

Previous work from our laboratory and others have shown that exposure of cancer cells to chemotherapeutic agents induces cellular senescence [27] [28], [29], suggesting that they may also induce SASP. Here we show that subjecting the melanoma cell line WM115 to treatment with a senescence-inducing drug doxorubicin, not only induced expression of previously reported components of SASP, but also that of various Wnt ligands. As shown in Figure 1A, drug-treatment induced a dose dependent inhibition of cell proliferation, with virtually no change in growth after 3 days in cells treated with the drug at 10^−6^ M. Concomitantly expression of p53, the cell cycle inhibitor p21/WAF1 and genes associated with EMT were increased. However cyclin D levels decreased in support of the observed reduction in cell proliferation (Fig. 1B).The results suggest that doxorubicin induces both EMT and senescence. Enhanced staining for the senescence-associated beta galactosidase (SA-beta Gal) was also observed particularly at 1 µM concentration of doxorubicin (Fig. 1C), indicating that the treated cells were in fact undergoing senescence. The expression profile of contributors of SASP, determined by quantitative PCR (Fig. 1D), shows that several of previously reported factors [6] were induced in response to doxorubicin. Interestingly, various members of the Wnt family were expressed in parallel with SASP (Fig. 1D). Drug-induced expression of Wnt ligands was also observed in other cells lines representing neuroblastoma (SKN-SH), melanoma (WM226) and colon cancer (SW480), indicating that this phenomenon may be valid for more than one cell line (Figure S1). Based on this, the signaling pathway leading to transactivation of β-catenin may also be induced as a result of cellular exposure to this drug.

### Activation of Wnt Signaling and Enhanced Expression of EMT Markers Accompany Drug-induced SASP

**Figure 2:**
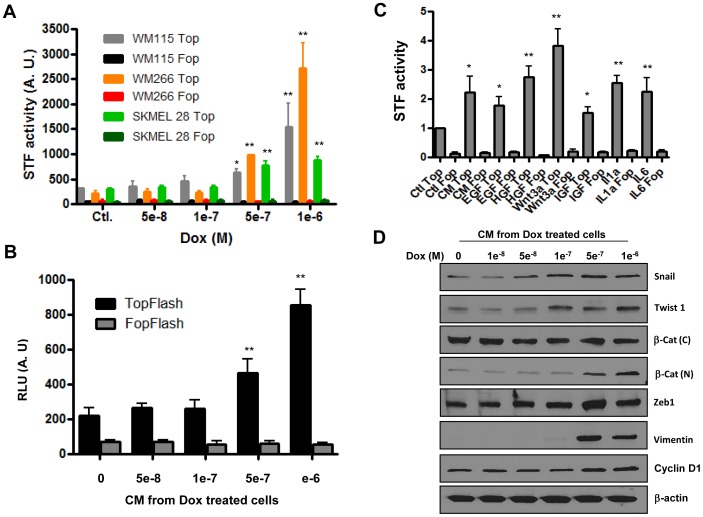
Wnt signaling activation and expression of EMT markers in association with SASP. Panel A. β-catenin transactivation, measured by the Super-Topflash luciferase reporter system (STF) in three melanoma cell lines exposed or not to increased concentrations of doxorubicin. The FopFlash construct containing mutated TCF binding sites was used to define the specificity of doxorubicin’s action. Panel B. Effect of conditioned media from cells pre-treated with increasing concentrations of doxorubicin on TopFlash and FopFlash activities in naïve cells (not previously exposed to the drug). Panel C. Effect of individual SASP components on β-catenin transactivation determined by measure of STF activity. Data in panels A, B and C represents the average of three determinations ±SE. Statistical significance is shown for drug-treated cells versus control (*p<0.05, **p<0.001). Panel D. Western blots showing expression of EMT genes in WM 115 cells incubated with conditioned medium from their senescent counterparts. β-actin is used as a loading control. β-cat (c): β catenin in the cytoplasm, β-cat (N): β-catenin in the nucleus.

To determine if the Wnt signaling pathway was activated in response to drug induced SASP, we measured beta catenin transactivation by using the TopFlash luciferase reporter construct containing several DNA binding elements for the TCF/LEF transcription complex [23]. To verify transfection efficiency, we used the same DNA construct in which the luciferase was replaced by EGFP and found that about 80% of cells were transfected (Figure S2). As shown in Figure 2A, exposure of melanoma cell lines to doxorubicin induced luciferase activity in a dose dependent manner, with a significantly higher level at the senescence inducing concentration of 10^−6^M. Of note, the FopFlash activity was not affected by this treatment, reflecting the specificity towards β-catenin/TCF activation. Similar findings were obtained with other drugs such as the proteasome inhibitor Bortezomib, and the chromatin modifier Belinostat, and in other cancer cell lines including neuroblastoma, colon cancer and breast cancer exposed to doxorubicin (Figure S3), suggesting that this may represent a general phenomenon. Considering the well established notion that activation of the Wnt pathway plays key roles in cancer progression and relapse, the data presented here suggests that *in vitro* testing of chemotherapeutic agents for ability to induce this pathway may be useful in decision making on whether or not to use these agents in cancer patients.

To investigate the possibility of a paracrine influence by senescent cancer cells on their neighbors, we tested whether conditioned medium (CM) from drug-treated cells could activate the Wnt signaling pathway in cells not previously exposed to the drug. As shown in Figure 2B, the TopFlash and not FopFlash activity was induced by CM from cells treated with senescence-inducing drug concentrations and not by the one from control non-treated cells. Of note, both Wnt and non-Wnt ligands such as growth factors and certain cytokines were able to induce β-catenin activation (Fig. 2C), supporting the central role of this pathway in mediating the action of SASP components. Since tumors are generally heterogeneous, the data suggest that drug-induced senescence in sensitive cells may cause secretion of factors that activate Wnt signaling in neighboring non-senescent cells, and thus, enhance their ability to migrate and survive in a toxic environment. In agreement with this, nuclear β-catenin levels increased and a number of its target genes including cyclin D1, Zeb1 and vimentin, also known for their role in tumor proliferation and invasion [30], [31], were induced upon exposure of naïve cells to CM from their senescent counterparts (Figure 2D).

### Implication of β-catenin Destruction Complex in Mediating the Action of SASP

**Figure 3:**
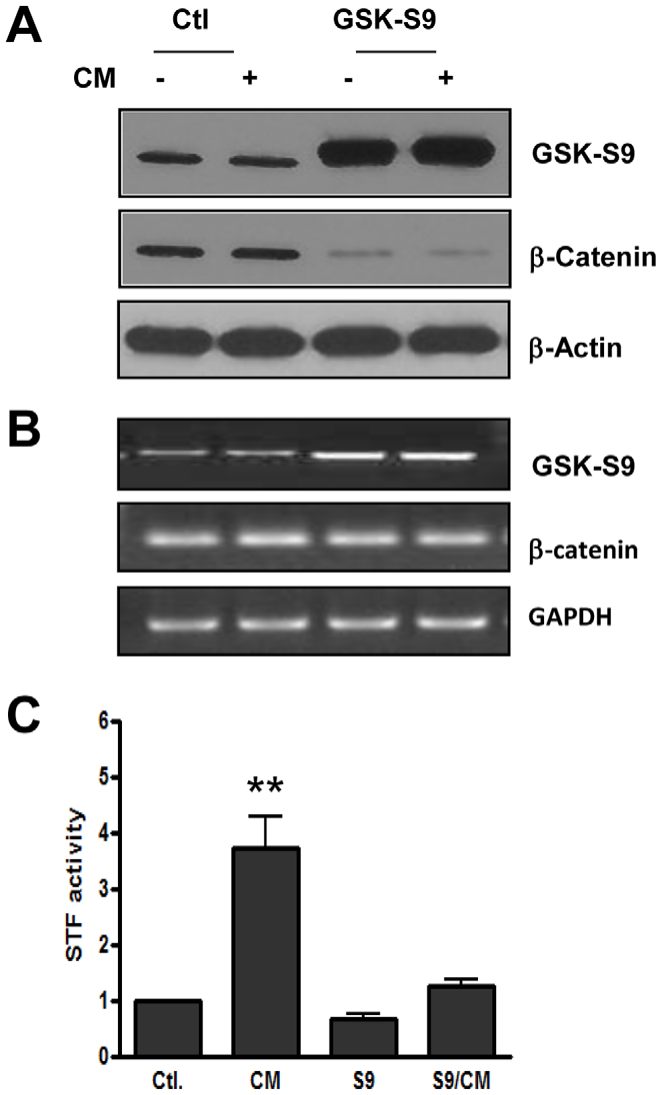
Role of GSK3-associated β-catenin degradation complex in mediating β-catenin transactivation by SASP. Panel A. Effect of enhanced GSK 3β activity on β-catenin integrity. 293 cells were transfected with a constitutively active form of GSK 3β (GSK3-S9), and expression levels of this enzyme and as well as β-catenin determined by western blot. β-actin is used here as a loading control. Panel B. RT-PCR analysis showing expression levels of GSK 3β, β-catenin and GAPDH in transfected and non-transfected cells as described in panel A. Panel C. Effect of GSK 3β activation on SASP induced β-catenin transactivation. GSK3-S9 transfected and non-transfected cells were incubated in the absence or the presence of CM from those treated with senescence inducing concentration of doxorubicin. STF activity was measured after 24 h. The data represents the average of three determinations ±SE (**p<0.001).

The observation that growth factors with separate mechanisms of action induce β-catenin transactivation (Fig. 2C and [32], [33]) suggests that a common mechanism mediating the actions of these molecules might exist. We suspected that BCDC may represent a common target of SASP components since, as mentioned above, this complex is known to integrate the action of several signaling pathways. We investigated a potential role of BCDC in this process by overexpressing a constitutively active form of GSK 3β and analyzing its effects on β-catenin integrity and activity. In this mutant enzyme, referred to here as GSK 3β-S9, serine S9 that is subject to phosphorylation-mediated inactivation of the wild type enzyme was replaced with alanine. As shown in Figure 3 A, overexpression of GSK 3β-S9 caused a strong decrease in total β-catenin levels, which apparently was not due to diminished expression of this gene as shown by RT-PCR (Fig. 3B), but likely to enhanced degradation of the corresponding protein. The data also show that β-catenin transactivation was dramatically reduced in the GSK 3β-transfected cells when exposed to CM from the drug-treated counterparts (Fig. 3C), suggesting that GSK 3β and thus the associated degradation complex (BCDC) may play important roles in mediating the action of SASP, and therefore represent suitable targets to suppress this phenomenon.

### Targeting BCDC to Inhibit the Activity of SASP

**Figure 4:**
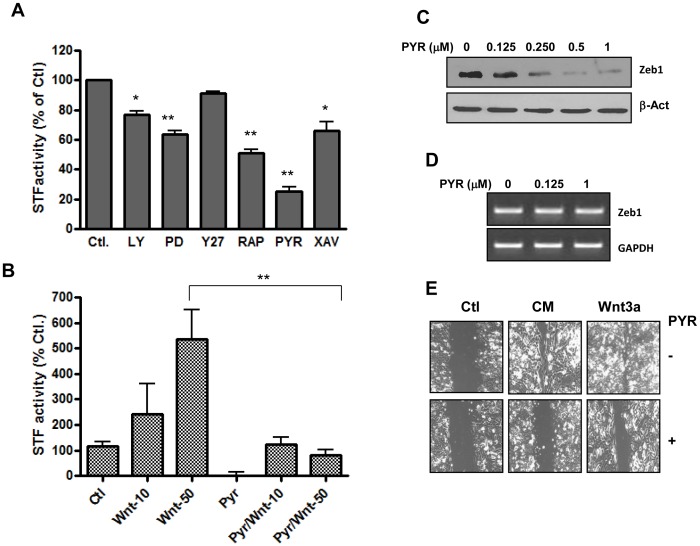
Targeting BCDC inhibits β-catenin transactivation, EMT and cell migration. Panel A. Effect of direct and indirect activators of the BCDC on β-catenin transactivation as measured by STF activity. STF transfected cells were pre-treated with the indicated effectors for 2 hrs and then exposed to CM from those pre-treated with senescence-inducing doxorubicin concentration for an additional 24 h, followed by measure of STF activity. LY: PI3 kinase inhibitor. (LY294002), PD: MAP kinase inhibitor (PD98059), Y27: Rho kinase inhibitor (Y27632), RAP: mTOR inhibitor (rapamycin). XAV: Tankyrase inhibitor (XAV939), and PYR: casein kinase 1 activator (Pyrvinium). Panel B. Effect of Pyrvinium (PY) on Wnt3a- mediated activation of TopFlash. Cells were exposed for 24 hours to Wnt 3a at 10 or 50 ng/ml (Wnt-10 and Wnt-50 respectively), in the absence or the presence of pyrvinium (PYR at 500 nM). Data in panels A and B represents average of three determinations ±SE. Statistical significance is shown in panel A for drug-treated cells versus control, and in panel B between Wnt-50+PYR compared to Wnt-50-treated cells (*p<0.05, **p<0.001). Panels C and D. Effect of cellular exposure to PYR for 24 h on the expression of Zeb1 at the protein (Western blot) and the mRNA level (RT-PCR). Panel E. Effect of PYR (500 nM) on cell migration as determined by the monolayer scratch assay described in the method section.

To determine if targeting BCDC affects the action of SASP, a series of small molecules known to act either directly or indirectly to regulate the activity of BCDC were analyzed for their ability to inhibit β-catenin transactivation and the action of SASP on cell invasion and resistance to therapy. As shown in Figure 4A, the Topflash activity was reduced by approximately 30% in the presence of the PI3kinase inhibitor, 40% by the MAP kinase inhibitor, 50% by the mTOR inhibitor and 35% by the tankyrase inhibitor XAV939. Except for XAV939 which inhibits a poly (ADP-ribose) polymerase responsible for Axin degradation [34], most of the tested compounds inhibit kinases upstream of GSK 3β. Therefore, they act as indirect activator of BCDC, which may explain their relatively modest effects in the β-catenin transactivation assay. By contrast, the activity of TopFlash was inhibited by about 80% in the presence of pyrvinium, a recently described direct activator of BCDC [35].

Validation of pyrvinium’s suppressive effect against Wnt3a-mediated induction of β-catenin transactivation was carried out in our cellular model (Figure 4B). Its effect on the EMT pathway was assessed by measure of Zeb1 expression and cell migration. Western blot analysis (Fig. 4C) indicated that Zeb1 levels were strongly reduced in the presence of this drug, likely due to proteolysis since the mRNA levels of this gene were not affected (Fig. 4D). Pyrvinium was also found to regulate cell migration measured using the well established monolayer scratch assay [36]. As shown in Figure 4E, cellular exposure to conditioned medium (CM) from cells exposed to 10^−6^M doxorubicin, or to Wnt3a (50 ng/ml), resulted in accelerated cell migration when compared to control, whereas the addition of pyrvinium delayed this process. The data suggest that BCDC activators may be useful in order to suppress the pro-migratory action of chemotherapy-induced SASP.

**Figure 5:**
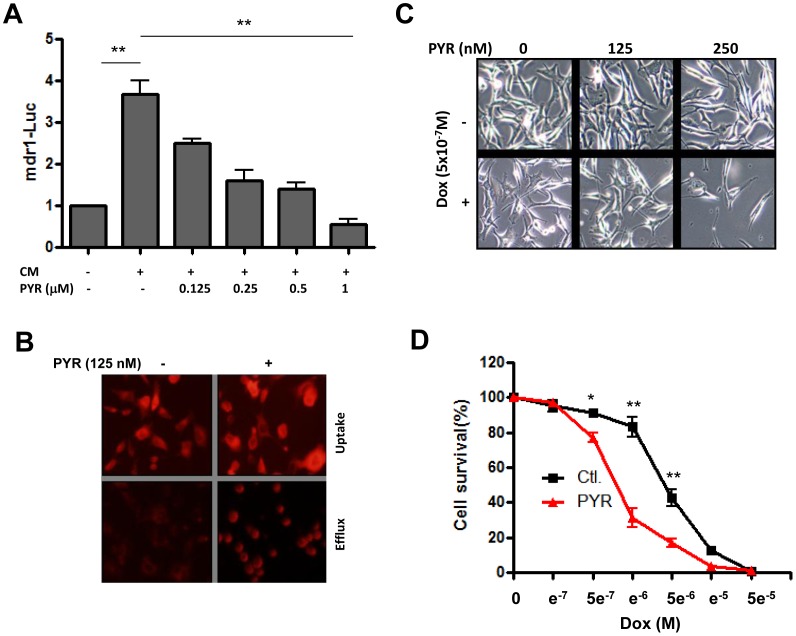
Effect of pyrvinium on SASP-induced expression of mdr1 gene and resistance to doxorubicin. Panel A. Pyrvinium was added at the indicated concentrations to 293 cells transfected with the PGL3-mdr1-luciferase (mdr1-Luc) and then incubated with CM from those treated with senescence inducing concentration of doxorubicin. After 24 h, the luciferase activity was measured and plotted as a ratio of luminescence measured in cells transfected with the mdr1-Luc versus those transfected with the PGL3-luciferase. Panel B. Effect of pyrvinium on rhodamine 123 uptake (1 hr) and efflux (2 hr). Panel C and D. Effect of pyrvinium (PYR) on cell viability in the melanoma cell line 266, determined morphologically and by MTT assay respectively. The data in panels A and D represent average ± SE of three determinations (*p<0.05, **p<00.1).

It has been shown that in addition to its effect on cell motility, SASP may also signal for resistance to therapy [7]. Based on this, we determined whether pyrvinium affects SASP-induced expression of the multidrug resistance gene *mdr1*, and as shown in Fig. 5A, this treatment strongly reduced the luciferase activity associated with the promoter of this gene. In addition, drug efflux activity of the *mdr1* gene product (P-glycoprotein) was also inhibited by pyrvinium in cells exposed to SASP (Fig. 5B), and as a result, cell viability was reduced when doxorubicin is combined with pyrvinium, compared to cells treated with each drug separately (Fig. 5 C and 5D). The data shown in graph 5D indicates that in the presence of a senescence-inducing concentration of doxorubicin (10^−6^M), about 90% of cells survived (although they do not proliferate as shown in Figure 1). However, when this drug was combined with pyrvinium at 500 nM, only 30% of cells survived, suggesting that the drug combination induces cell death. Since many SASP components are considered as survival factors, inhibition of their effects by pyrvinium should facilitate doxorubicin induced apoptotic cell death. This may explain the strong decrease in cell survival in the presence of drug combination. The data is also in agreement with the previous finding [37] that pyrvinium enhances doxorubicin activity in a mouse model and provides a mechanistic explanation for it. Therefore, the use of direct activators of BCDC such as pyrvinium may represent an effective approach to suppress the action of chemotherapy-induced SASP on cancer cell invasion and resistance to drugs.

## Discussion

The present study addresses a paradigm by which chemotherapy-induced senescence in a subset of cancer cells could render the tumor as a whole more aggressive, rather than attenuating its severity. Historically, anti-tumor agents were identified based on their ability to inhibit cancer cell proliferation, with the ultimate goal of achieving irreversible growth arrest (senescence). However, due to cancer cell heterogeneity and differences in accessibility to drugs within the tumor mass, only a fraction of cells may undergo senescence. Consequently, the senescent cell population could signal through SASP, not only for enhanced survival of neighboring non-senescent cells, but also for their migration to distant sites. Our data support this concept and provide evidence for the central role of the β-catenin destruction complex in mediating the action of SASP and thus, as a potential target to suppress it. As shown in Figure 1 and Figure S1, drugs used to treat cancer were found to induce the expression of soluble factors known for their implication in metastasis and resistance to therapy. β-catenin transactivation, used as readout for the activity of SASP was also induced as a result of cancer cell exposure to these drugs (Fig. 2). More importantly, a causal relationship between this signaling pathway and secretion of SASP was demonstrated (Fig. 3). The key role of SASP in inter-cellular signaling between senescent and non-senescent cancer cells is demonstrated by the finding that culture medium (CM) from doxorubicin-treated cells induced β-catenin transactivation in those not previously exposed to the drug (Fig. 2C). Individual growth factors such as EGF, HGF, Wnt 3a and IGF, and cytokines IL6 and IL1 alpha were also able to induce TopFlash activity to different degrees, reinforcing the notion that the BCDC is not only a target for Wnt ligands but also for other growth factors, cytokines and related signaling pathways.

Possible consequences of drug-induced SASP are enhanced expression of EMT genes in both senescent and non-senescent cells (Figures 1B and 2D). While this should not have any significant effect on senescent cells since they are not proliferating, exposure to CM from these cells will likely accelerate migration of their non-senescent neighbors (Fig. 4E). Based on this, the likelihood of a given drug to induce SASP should be taken into consideration before inclusion in therapeutic regimen. Alternatively, approaches to inhibit SASP could be used to improve the efficacy of anticancer drugs. The finding that pyrvinium, an activator of the β-catenin destruction complex, was effective in inhibiting cell migration (Fig. 4) represents a proof of principle for this. Pyrvinium is an FDA approved drug for anthelminthic use. It is currently under evaluation for anticancer activity, thanks to recent findings regarding its inhibitory effect against Wnt signaling. In these studies, pyrvinium was found to act as a direct activator of the casein kinase1 alpha, a key element of BCDC [35]. It has also been shown to exert anti-proliferative effects on cancer cells *in vitro* and *in vivo* [38], however its effect on metastasis-related processes has not yet been reported. In this regard, the data presented here suggest that pyrvinium or other putative activators of BCDC may represent useful therapy adjuvants to suppress cancer progression.

Besides metastasis, another cause of cancer recurrence is the development of drug resistance. Over the years, we come to realize that cancer cells have the ability to adapt and ultimately escape the toxicity of virtually any drug tested so far. The underlying mechanisms are numerous and well described, however the initial steps leading to development of resistance are not yet understood. In this regard, the finding that SASP induce the development of drug resistance [7] provides a unique opportunity for exploring the causes of this phenomenon in order to prevent its onset. Our data (Fig. 5) suggest that this may be possible since pyrvinium inhibits expression of the *mdr1* gene (Fig. 5A) as well as the drug efflux activity associated with the corresponding gene product (Fig. 5B). As a result, cellular response to doxorubicin was enhanced when these cells were cultivated in the presence of pyrvinium (Fig. 5C and 5D). The observed increase in cell death in the presence of the drug combination (Fig. 5D) may be explained by the fact that, since many SASP components are considered as survival factors, they will likely suppress doxorubicin-induced apoptosis. However inhibition of their effects by pyrvinium should facilitate doxorubicin induced apoptotic cell death.

Overall, these findings are of particular interest considering the fact that most existing drug resistance-reversing agents act by inhibiting the activity of drug transporters (i.e. P-glycoprotein). However, these agents often lack specificity and in many cases, inhibit other ions transport channels causing serious side effects [39]. In light of this, agents that suppress the expression of the *mdr1* gene may be more effective. Previous reports have shown that β-catenin induces expression of *mdr1* [21] and silencing of its partner TCF4 was effective in reversing drug resistance [40]. Our data is in support of these findings and introduce pyrvinium as potential pharmacological modulator acting at early stages to prevent the development of drug resistance. In conclusion, results from this study shed light on a novel mechanism by which certain chemotherapeutic agents, contrary to their intended use, may facilitate tumor metastasis and resistance to treatment. Based on this, the ability of drugs to induce SASP should be taken into consideration before inclusion in therapeutic regimens for cancer patients. Moreover, considering the central role that BCDC plays in mediating the action of drug-induced SASP, its targeting may improve the efficacy of existing and prospective anticancer agents.

## Supporting Information

Figure S1 **Doxorubicin-induced expression of Wnt ligands in other cell lines.** Neuroblastoma (SKN-SH), Melanoma WM 266, and colon cancer SW480 cells were incubated in the absence or presence of doxorubicin 1 µM for 24 hours and expression of Wnt ligands measured by QPCR as described in the Methods section. Data represent the average of three determination ±SE. Statistical significance is shown for drug-treated cells versus control (*p<0.05, **p<0.001).
(TIF)

Figure S2 **Transfection efficiency of TopFlash.** A DNA construct in which the TopFlash Luciferase is replaced by EGFP was used to transfect 293 cells under the same conditions used to transfect with the TopFlash construct (described in material and Method section).The cells were then visualized under phase contrast or fluorescence microscopy (left panel), and the percentage of transfected cells is graphed (right panel).
(TIF)

Figure S3 **β-catenin transactivation in different cell lines and in response to different drugs.** Panel A. Neuroblastoma (SKN-SH), colon Cancer SW480) and Breast cancer (MCF7) cell lines were transfected with the STF reporter and then exposed to the indicated concentrations of doxorubicin for 24 hours. The luciferase activity was then measured. Panels B and C. WM115 melanoma cells transfected with the STF reporter were exposed either to Belinostat (Panel B) or Bortezomib (Panel C) and luminescence was measured after 24 hrs. Values, normalized to control non-treated cells, represent the average of three determination ±SE. Statistical significance is shown for drug-treated cells versus control (*p<0.05, **p<0.001).
(TIF)
