# Supplementary figures and images for: Role of the Beta Catenin Destruction Complex in Mediating Chemotherapy-Induced Senescence-Associated Secretory Phenotype
Source: PLoS One. 2012 Dec 18;7(12):e52188. doi: 10.1371/journal.pone.0052188 (PMC3525570; doi:10.1371/journal.pone.0052188)

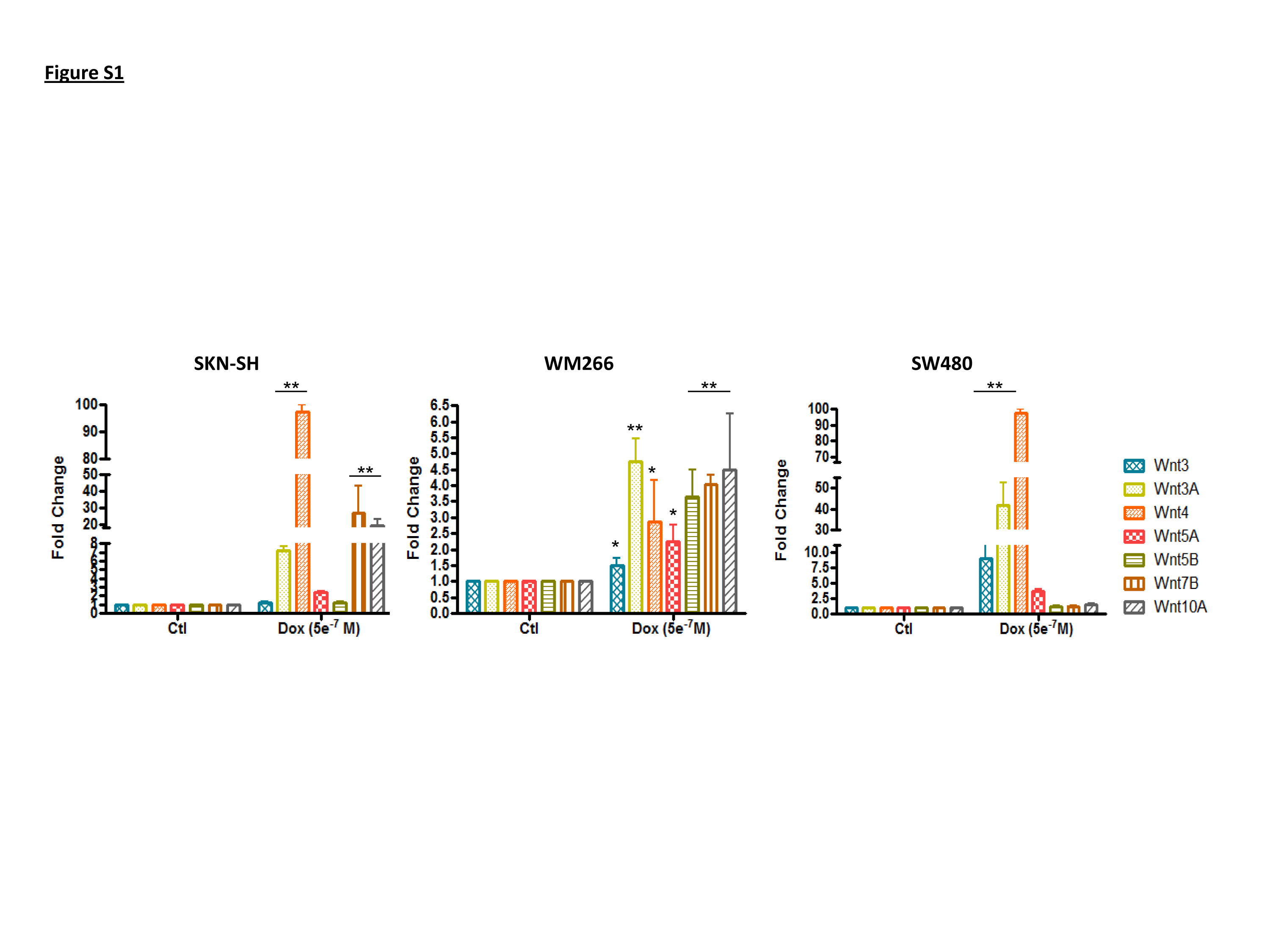

Supplement: Figure S1 — Doxorubicin-induced expression of Wnt ligands in other cell lines. Neuroblastoma (SKN-SH), Melanoma WM 266, and colon cancer SW480 cells were incubated in the absence or presence of doxorubicin 1 µM for 24 hours and expression of Wnt ligands measured by QPCR as described in the Methods section. Data represent the average of three determination ±SE. Statistical significance is shown for drug-treated cells versus control (*p<0.05, **p<0.001). (TIF) [file pone.0052188.s001.tif]

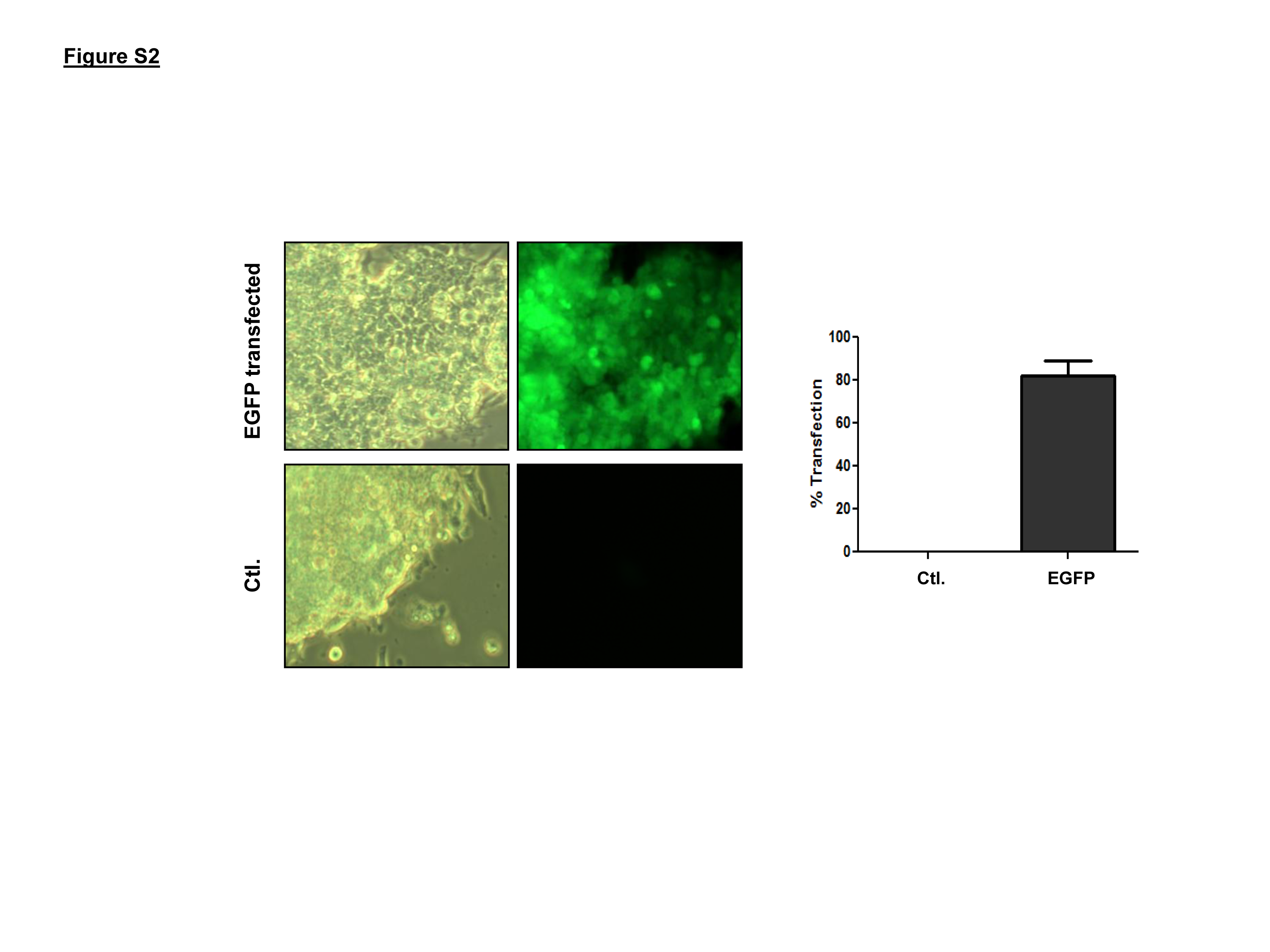

Supplement: Figure S2 — Transfection efficiency of TopFlash. A DNA construct in which the TopFlash Luciferase is replaced by EGFP was used to transfect 293 cells under the same conditions used to transfect with the TopFlash construct (described in material and Method section).The cells were then visualized under phase contrast or fluorescence microscopy (left panel), and the percentage of transfected cells is graphed (right panel). (TIF) [file pone.0052188.s002.tif]

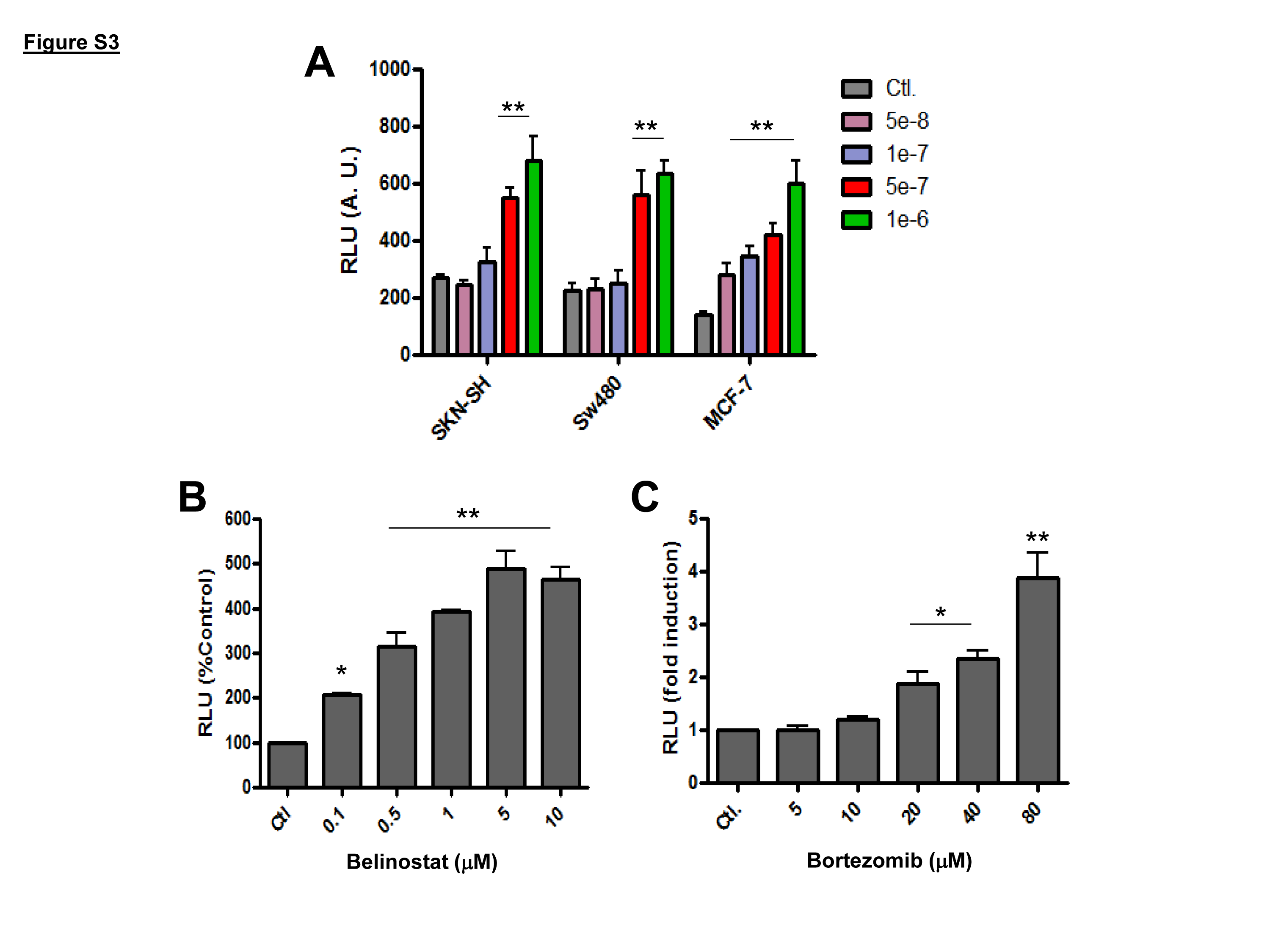

Supplement: Figure S3 — β-catenin transactivation in different cell lines and in response to different drugs. Panel A. Neuroblastoma (SKN-SH), colon Cancer SW480) and Breast cancer (MCF7) cell lines were transfected with the STF reporter and then exposed to the indicated concentrations of doxorubicin for 24 hours. The luciferase activity was then measured. Panels B and C. WM115 melanoma cells transfected with the STF reporter were exposed either to Belinostat (Panel B) or Bortezomib (Panel C) and luminescence was measured after 24 hrs. Values, normalized to control non-treated cells, represent the average of three determination ±SE. Statistical significance is shown for drug-treated cells versus control (*p<0.05, **p<0.001). (TIF) [file pone.0052188.s003.tif]
